# Multiantenna Relay Beamforming Design for QoS Discrimination in Two-Way Relay Networks

**DOI:** 10.1155/2013/306423

**Published:** 2013-12-10

**Authors:** Ke Xiong, Yu Zhang, Dandan Li, Chih-Yung Chang, Zhangdui Zhong

**Affiliations:** ^1^School of Computer and Information Technology, State Key Laboratory of Rail Traffic Control and Safety, Beijing Jiaotong University, Beijing 100044, China; ^2^Department of Electronic Engineering, Tsinghua University, Beijing 100084, China; ^3^Department of Computer Science and Information Engineering, Tamkang University, New Taipei City 25137, Taiwan

## Abstract

This paper investigates the relay beamforming design for quality of service (QoS) discrimination in two-way relay networks. The purpose is to keep legitimate two-way relay users exchange their information via a helping multiantenna relay with QoS guarantee while avoiding the exchanged information overhearing by unauthorized receiver. To this end, we propose a physical layer method, where the relay beamforming is jointly designed with artificial noise (AN) which is used to interfere in the unauthorized user's reception. We formulate the joint beamforming and AN (BFA) design into an optimization problem such that the received signal-to-interference-ratio (SINR) at the two legitimate users is over a predefined QoS threshold while limiting the received SINR at the unauthorized user which is under a certain secure threshold. The objective of the optimization problem is to seek the optimal AN and beamforming vectors to minimize the total power consumed by the relay node. Since the optimization problem is nonconvex, we solve it by using semidefinite program (SDP) relaxation. For comparison, we also study the optimal relay beamforming without using AN (BFO) under the same QoS discrimination constraints. Simulation results show that both the proposed BFA and BFO can achieve the QoS discrimination of the two-way transmission. However, the proposed BFA yields significant power savings and lower infeasible rates compared with the BFO method.

## 1. Introduction

This paper considers the relay beamforming design to achieve the quality of service (QoS) discrimination between the legitimate users and unauthorized receiver in two-way relay networks, where the system is required to design with such ability; namely, the system is capable of guaranteeing the required communication QoS for the legitimate users meanwhile avoiding the information overhearing by the unauthorized receivers (eavesdroppers). This kind of problems actually can be found in many wireless applications, because of the broadcast nature of wireless medium. For example, the reception performance discrimination is often required between paid and unpaid users in TV broadcast systems, between the members and nonmembers of some organizations in information sharing, and also between authorized users and eavesdropping receivers in secure communications [1].

Traditionally, such QoS discrimination problem was addressed with the employment of application level cryptography and user authentication mechanisms, but recent developments in physical layer secrecy [2] show that this problem can also be effectively handled in the physical layer by exploiting the difference of the fading channels among legitimate and unauthorized users [1].

As for secure communication on QoS discrimination, some works can be found in the literature; see, for example, [1, 3–5], where in [3], the secure communication over broadcast channel was investigated, while it was assumed that the transmitter can broadcast signals with a nonzero coding rate to the legitimate users without any information being eavesdropped by unauthorized users if the mutual information between the transmitter and the legitimate users is higher than that between transmitter and unauthorized users. This physical layer paradigm in information theory has inspired several recent research efforts; see, for example, [4, 5], where the physical layer secrecy was enhanced via signal processing techniques. It is deserved to be stressed that some of existing works began to consider using the spatial degree of freedom with deploying multiple antennas on the transmitters to achieve and enhance the secrecy for physical layer communications. Specifically, with the use of multiple antennas at the transmitter, beamforming techniques can be adopted to effectively discriminate the reception performances between the legitimate and unauthorized users [4, 5].

Most recently, the authors in [6] proposed an interesting and insightful beamforming scheme where an artificial noise (AN) is purposely added in the transmitted signal for raising the interference level at eavesdroppers. With the channel state information (CSI) known at the transmitter, the AN-aided method may allocate the vector of AN in an orthogonal space of the transmitter-to-legitimate-user's channel in a spatially uniform fashion [7, 8]. By exploiting the CSIs of the unauthorized user, the AN energies may be concentrated on the eavesdroppers' directions to make the QoS discrimination even more effective in practice.

Due to the efficiency of AN-based beamforming design, it has attracted much attention; see, for example, [7–10]. However, most of them considered the joint AN and source beamforming design for one-way transmission wireless systems, where the works in [7, 8] addressed the problem in one-hop broadcast channels and those in [9, 10] addressed the problem for one-way two-hop relay channels.

As the two-way relay transmission is considered as a promisingly applicable transmission model in many wireless systems [11, 12], in this paper, we focus on the joint AN and beamforming design for two-way relay networks.

The contributions of our work are summarized as follows. *Firstly*, we propose a physical layer scheme to achieve QoS discrimination by using AN together with relay beamforming design (BFA) for two-way relay networks. To the best of our knowledge, only two works have investigated the AN-based beamforming design for two-way relay networks, where in both [11, 12] the two-way relay networks with single-antenna relays were considered. Different from existing works, we consider a two-way relay system with a multiantenna relay node. Our goal is to jointly optimally design the AN and beamforming vectors to discriminate the receiving performance between the legitimate and unauthorized users. *Secondly*, we formulate the joint beamforming design into an optimization problem. Our goal is to seek for the jointly optimalized AN and beamforming vectors to minimize the total power consumed by the relay node under the QoS discrimination constraints. Since the optimization problem is nonconvex, we solve it with semidefinite program (SDP) relaxation. *Thirdly*, for comparison, the optimal relay beamforming without using AN method (BFO) is also studied for two-way relay networks. Based on this, extensive simulation results are presented, which show that both the proposed BFA and BFO can realize the QoS discrimination for the two-way relay transmissions. However, the proposed BFA yields significant power savings than BFO.

The rest of this paper is organized as follows.  Section 2 introduces the system model.  Section 3 describes the proposed BFA, where the joint AN and beamforming vectors design is formulated into an optimization problem and then relaxed into a convex optimization problem by using the SDR method.  Section 4 investigates the optimal beamforming design for BFO for compassion.  Section 5 presents some simulation results to evaluate the performance of the proposed method, and the paper is summarized with conclusions in Section 6.

## 2. System Model

**Figure 1:**
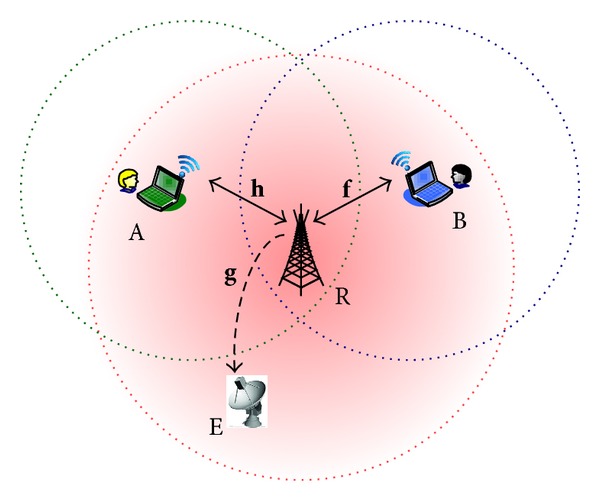
System model and the illustration of secure relay beamforming with artificial noise.

Consider a two-way relay transmission scenario as shown in Figure 1, where two sources, A and B, exchange their information via a relay node R with *K* antennas, in the presence of an eavesdropper E. We assume that A and B are too far away from each other so that there is no direct link between them. Thus, all information exchange between A and B is helped by the assistant relay R.

It is assumed that the two sources are equipped with single antenna and all channel matrices of the links are known to the transceivers. It is also assumed that the eavesdropper's CSI can be obtained when the eavesdroppers are active in the network [10]. This assumption is applicable in wireless systems, particularly in the multicast and unicast coexisted networks, where terminals play dual roles as legitimate receivers for some signals and eavesdroppers for others.

Half-duplex constraint is considered, so that two phases, that is, the Multiple Access (MA) phase and the Broadcast (BC) phase, are involved to complete a round of information exchange between A and B. In the MA phase, A and B send their signal to the relay node simultaneously. AF protocol is employed, so in the BC phase, relay amplifies the received signals and then broadcasts them through its *K* antennas to A and B simultaneously. As A and B know their own transmitted signals, the self-interference is able to be canceled and the desired information can be extracted from the received mixed signals. Note that, due to the broadcast nature of wireless links, the eavesdropper E which resides in the system may overhear the information exchange between A and B. Here we assume that E is not within the coverage areas of both A and B but within the service range of relay R. In this case, all signals transmitted from R for A and B may be collected by E.

To keep the QoS discrimination for such a two-way relay transmission, the relay node should (1) guarantee the information exchange quality between A and B and, at the same time, (2) prevent the information leakage to the eavesdropper to keep the QoS discrimination. In order to meet these two goals simultaneously, similar to many secure physical layer system design, see, for example, [7], we adopt the received SINR as a measurement. Specifically, the received SINR at each source node should be kept above a predefined QoS threshold to keep the quality of the two-way relay information exchange, while the received SINR at E should be limited below a predefined security threshold, avoiding the information overhearing by the eavesdropper.

Let **h** = [*h*_1_,…, *h*_*K*_], **f** = [*f*_1_,…*f*_*K*_], and **g** = [*g*_1_,…*g*_*K*_] denote the quasistationary flat-fading channel coefficient vectors between A, B, E, and the relay node, respectively. To keep the QoS discrimination for the two-way relay network, we propose a beamforming design method by using artificial noise.

**Figure 2:**
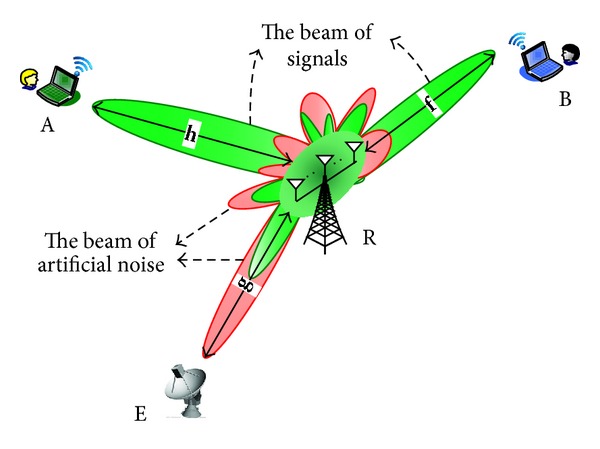
Relay beamforming with artificial noise.

Our basic idea is that (as shown in Figure 2), by proper joint AN and relay beamforming design, the beam pattern of artificial noise can be mainly directed to the eavesdropper, which may effectively interfere with the unauthorized user's reception, while the beam pattern of the signals for the two-way relay users can be mainly directed to the two sources. By doing so, the QoS discrimination for the two-way relay network is able to be enhanced.

Let *γ*_A_ and *γ*_B_ be predefined QoS thresholds, for A and B, respectively, and let *γ*_E_A__ and *γ*_E_B__ be the predefined secure thresholds at E, for A and B, respectively. Then, an optimization framework of our joint AN and relay beamforming design can be mathematically given by

(1) min⁡w,Σ PR+PANs.t. SINRA≥γA,    SINRB≥γB,    SINREA≤γEA,    SINREB≤γEB,

where *P*_R_ is total power of the relay beamforming vectors and *P*_AN_ is total power of the artificial noise. SINR_A_ and SINR_B_ are the end-to-end received SNR at A and B, respectively. SINR_E_A__ and SINR_E_B__ are the received SNR at E for the signal transmitted from B and A and the signal transmitted from B and A, respectively. **w** and Σ represent the relay beamforming vector and artificial noise vector, respectively.

## 3. Optimal Relay Beamforming with Artificial Noise 

This section presents the proposed BFA method and then designs the optimal AN and beam vectors for it.

### 3.1. BFA

In the MA phase of BFA, A and B send their information to the relay node simultaneously. Thus, the received signals at the relays and at E can be, respectively, given by

(2) $$\begin{array}{c} y_{\text{R}}=\sqrt{P_{\text{A}}}h_{\text{R}}x_{\text{A}}+\sqrt{P_{\text{B}}}f_{\text{R}}x_{\text{B}}+n_{\text{R}}, \end{array}$$

where **y**_R_ is a *K* × 1 complex vector of the received signal at the *K* antennas at R and *y*_E_ is the received signal at E in the MA phase. *P*_A_ and *P*_B_ denote the transmit power of A and B, respectively. **n**_R_ is a *K* × 1 complex vector of Additive White Gaussian Noise (AWGN) at the relay.

In the BC phase, the relay amplifies the received signal for the *i*th antenna by a complex beamforming weight *w*_*i*_. Thus the beam for the amplified signals can be expressed as **Ω** **y**_R_. Here, we adopt the artificial noise method [6], where the relay transmits artificial noise (interference) to mask the concurrent transmission of information bearing signal to the eavesdroppers. Let *ε* be the *K* × 1 artificial noise vector. Then, the transmit signal vector **x** at the relay node can be expressed as

(3) $$\begin{array}{c} x_{\text{R}}^{\left(\text{RFA}\right)}=\Omega y_{\text{R}}+\varepsilon . \end{array}$$

Note that the design artificial noise follows the zeros-mean complex Gaussian distribution with covariance matrix Σ⪰0. Thus, the signal received at A, B, and E can be expressed as

(4) yA(BFA)=hRTx(BFA)+nA=PAhRTΩhRxA︸self-interference+PBhRTΩfRxB︸desired signal +hRTΩnR+nA︸noise+hRTε︸AN,yB(BFA)=fRTx(BFA)+nB=PAfRTΩhRxA︸desired signal+PBfRTΩfRxB︸self-interference +fRTΩnR+nB︸noise+fRTε︸AN,yE(BFA)=gRTx(BFA)+nB=PAgRTΩhRxA︸desired signal+PBgRTΩfRxB︸self-interference +gRTΩnR+nE︸noise+gRTε︸AN,

respectively. In terms of (4), the end-to-end received SINRs at A and B can be given by

(5) $$\begin{array}{c} {\text{SINR}}_{\text{A}}^{\left(\text{BFA}\right)}=\frac{P_{\text{B}}w^{H}G_{\text{AB}}w}{\sigma^{2}w^{H}D_{\text{A}}w+\mathrm{tr}\left(H_{\text{A}}\Sigma \right)+\sigma^{2}},\\ {\text{SINR}}_{\text{B}}^{\left(\text{BFA}\right)}=\frac{P_{\text{A}}w^{H}G_{\text{AB}}w}{\sigma^{2}w^{H}D_{\text{B}}w+\mathrm{tr}\left(H_{\text{B}}\Sigma \right)+\sigma^{2}}, \end{array}$$

where **H**_A_ = **h**_R_ **h**_R_^*H*^, **H**_B_ = **f**_R_ **f**_R_^*H*^, and

(6) $$\begin{array}{c} G_{\text{AB}}=\mathrm{diag}\left(h_{\text{R}}\right)f_{\text{R}}{\left(\mathrm{diag}\left(h_{\text{R}}\right)f_{\text{R}}\right)}^{H},\\ D_{\text{A}}=\mathrm{diag}\left(h_{\text{R}}\right){\left(\mathrm{diag}\left(h_{\text{R}}\right)\right)}^{H},\\ D_{\text{B}}=\mathrm{diag}\left(f_{\text{R}}\right){\left(\mathrm{diag}\left(f_{\text{R}}\right)\right)}^{H},\\ w={\left[w_{1},w_{2},\ldots w_{K}\right]}^{H}. \end{array}$$

**w** is the beam vector which is required to design. By using some signal detection methods, E tries to decode *x*_A_ and *x*_B_. The received SINR at E for decoding *x*_A_ and *x*_B_ can be, respectively, given by

(7) $$\begin{array}{c} {\text{SINR}}_{{\text{E}}_{\text{A}}}^{\left(\text{BFA}\right)}=\frac{P_{\text{A}}w^{H}G_{\text{AE}}w}{P_{\text{B}}w^{H}G_{\text{BE}}w+\sigma^{2}w^{H}D_{\text{E}}w+\mathrm{tr}\left(F_{\text{E}}\Sigma \right)+\sigma^{2}},\\ {\text{SINR}}_{{\text{E}}_{\text{B}}}^{\left(\text{BFA}\right)}=\frac{P_{\text{B}}w^{H}G_{\text{BE}}w}{P_{\text{A}}w^{H}G_{\text{AE}}w+\sigma^{2}w^{H}D_{\text{E}}w+\mathrm{tr}\left(F_{\text{E}}\Sigma \right)+\sigma^{2}}, \end{array}$$

where **F**_E_ = **g**_R_ **g**_R_^*H*^ and

(8) $$\begin{array}{c} G_{\text{AE}}=\mathrm{diag}\left(h_{\text{R}}\right)g_{\text{R}}{\left(\mathrm{diag}\left(h_{\text{R}}\right)g_{\text{R}}\right)}^{H},\\ G_{\text{BE}}=\mathrm{diag}\left(f_{\text{R}}\right)g_{\text{R}}{\left(\mathrm{diag}\left(f_{\text{R}}\right)g_{\text{R}}\right)}^{H},\\ D_{\text{E}}=\mathrm{diag}\left(g_{\text{R}}\right){\left(\mathrm{diag}\left(g_{\text{R}}\right)\right)}^{H}. \end{array}$$

Based on the description above, we are going to find the jointly optimized **w** and Σ for BFA.

### 3.2. Optimal Beam Vectors Design for BFA

In this section, we design the jointly optimized beamforming vector **w** and artificial noise vector Σ for BFA to minimize the total transmit powers at the relay R. To meet the security requirement of the system, two constraints are considered.

Since

(9) $$\begin{array}{c} P_{\text{R}}=E\left\{x_{\text{R}}^{\left(\text{PA}\right)}{\left(x_{\text{R}}^{\left(\text{PA}\right)}\right)}^{H}\right\}, \end{array}$$

according to (3), we can extend the expression for *P*_R_ as

(10) PR=E{xR(PA)(xR(PA))H}=PAwHDAw+PBwHDBw+σ2wHw+tr⁡(Σ),

where tr⁡(Σ) actually is the power of artificial noise. By substituting (5), (7), (9) and (10) into problem (1), then we have that

(11) min⁡w,Σ PAwHDAw+PBwHDBw+σ2wHw+tr⁡(Σ)s.t. PBwHGABwσ2wHDAw+tr⁡(HAΣ)+σ2≥γA   PAwHGABwσ2wHDBw+tr⁡(HBΣ)+σ2≥γB   PAwHGAEwPBwHGBEw+σ2wHDEw+tr⁡(FEΣ)+σ2≤γEA   PBwHGBEwPAwHGAEw+σ2wHDEw+tr⁡(FEΣ)+σ2≤γEB.

Since problem (11) is also nonconvex, by using the SDP relaxation method [13], problem (11) also can be relaxed to be convex. Let **W** = **w** **w** ^*H*^; problem (11) then can be relaxed as (12a). Consider

(12a)  min⁡W,Σ PAtr⁡(DAW)+PBtr⁡(DBW)+σ2tr⁡(W)+tr⁡(Σ)

(12b) s.t. σ2γAtr⁡(DAW)−PBtr⁡(GABW)+γAtr⁡(HAΣ)+σ2γA≤0

(12c) σ2γBtr⁡(DBW)−PAtr⁡(GABW)+γBtr⁡(HBΣ)+σ2γB≤0

(12d) PAtr⁡(GAEW)−σ2γEAtr⁡(DEW)−(σ2+PBtr⁡(GBEW)+tr⁡(FEΣ))γEA≤0

(12e) PBtr⁡(GBEW)−σ2(γEB−ηEB)tr⁡(DEW)−(σ2+PAtr⁡(GAEW)+tr⁡(FEΣ))×(γEB−ηEB)≤0

(12f)  W⪰0

(12g)  rank⁡(W)=1.

Following the SDP relaxation theory, the hard constraint rank⁡(**W**) = 1 also can be neglected and then the new relaxed problem is given by

(13) min⁡W,Σ PAtr⁡(DAW)+PBtr⁡(DBW)+σ2tr⁡(W)+tr⁡(Σ)s.t. (12b),(12c),(12d),(12e),(12f),

which is a convex SDP, and therefore can be efficiently solved to obtain the global optimum by the available solvers, for example, CVX [14].

It should be noted that, since the rank-one constraint is dropped in (12a), the optimal solution W* is not necessarily rank-one. Based on the rank reduction results for general SDPs, namely, Lemma  3.1 in [15], we can derive that rank⁡(W*) = 1 or rank⁡(W*) = 2. Therefore, if rank⁡(W*) = 1, the optimal beamforming vector w* can be retrieved from W* exactly. If rank⁡(W*) = 2, Gaussian randomization method [12] can be applied to obtain an approximated w*. Interestingly, the optimal solutions W* in our simulations are all rank-one, which means that w* can be retrieved from W* exactly.

## 4. Relay Beamforming without Artificial Noise (BFO)

### 4.1. BFA

In this subsection, we describe the BFO scheme. The process in the MA phase of BFA is the same as that of BFO, so we do not repeat the description of it again.

In the BC phase, the *i*th antenna amplifies the received signal by a complex beamforming weight *w*_*i*_. Thus, the processed signal vector at the relay node can be written as a *K* × 1 complex vector as follows:

(14) $$\begin{array}{c} x_{\text{R}}^{\left(\text{BFO}\right)}=\Omega {\text{y}}_{\text{R}}, \end{array}$$

where **Ω** = diag⁡([*w*_1_, *w*_2_,…*w*_K_]).

After this, the relay broadcasts the processed signals to A and B. So, the signals received at A, B, and E can be expressed as

(15) yA(BFO)=hRTx(BFO)+nA=hRTΩyR+nA=PAhRTΩhRxA︸self-interference+PBhRTΩfRxB︸desired signal+hRTΩnR+nA︸noiseyB(BFO)=fRTx(BFO)+nB=fRTΩyR+nB=PAfRTΩhRxA︸desired signal+PBfRTΩfRxB︸self-interference+fRTΩnR+nB︸noiseyE(BFO)=gRTx(BFO)+nEBC=gRTΩyR+nEBC=PAgRTΩhRxA+PBgRTΩfRxB+gRTΩnR+nEBC︸noise,

where *n*_A_, *n*_B_, and *n*_E_BC__ are the noise received at A, B, and E, respectively. Since A and B know their own transmitted signals, that is, *x*_A_ and *x*_B_, respectively, they can cancel the self-interference. Thus, in terms of (5) and (7), the end-to-end received SINR at A and B can be, respectively, given in

(16) $$\begin{array}{c} {\text{SINR}}_{\text{A}}^{\left(\text{BFO}\right)}=\frac{P_{\text{B}}w^{H}G_{\text{AB}}w}{{\sigma^{2}w^{H}D_{\text{A}}w+\sigma }^{2}}\\ {\text{SINR}}_{\text{B}}^{\left(\text{BFO}\right)}=\frac{P_{\text{A}}w^{H}G_{\text{AB}}w}{{\sigma^{2}w^{H}D_{\text{B}}w+\sigma }^{2}}. \end{array}$$

For E, it collects the signals in both phases; we assume that the MRC is used at E to extract the desired signals. Therefore, the received SINR for the signals transmitted from A and B at E can be given, respectively, by

(17) $$\begin{array}{c} {\text{SINR}}_{{\text{E}}_{\text{A}}}^{\left(\text{BFO}\right)}=\frac{P_{\text{A}}w^{H}G_{\text{AE}}w}{P_{\text{B}}w^{H}G_{\text{BE}}w+\sigma^{2}w^{H}D_{\text{E}}w+\sigma^{2}}\\ {\text{SINR}}_{{\text{E}}_{\text{B}}}^{\left(\text{BFO}\right)}=\frac{P_{\text{B}}w^{H}G_{\text{BE}}w}{P_{\text{A}}w^{H}G_{\text{AE}}w+\sigma^{2}w^{H}D_{\text{E}}w+\sigma^{2}}. \end{array}$$

### 4.2. Optimal Beam Vector Design for BFO

Compared with the *P*_R_ of BFA, the *P*_R_ of BFO has only one term, that is, without tr⁡(Σ). Similarly to the analysis for BFA,

(18) PR=PR(BFO)=E{xR(BFO)(xR(BFO))H}=PAwHDAw+PBwHDBw+σ2wHw.

By substituting (18), (16), (17), and *P*_AN_ = 0 into (7), we have that

(19) min⁡w PAwHDAw+PBwHDBw+σ2wHws.t. PBwHGABwσ2wHDAw+σ2≥γA   PAwHGABwσ2wHDBw+σ2≥γB   PAwHGAEwPBwHGBEw+σ2wHDEw+σ2≤γEA   PAwHGAEwPBwHGBEw+σ2wHDEw+σ2≤γEA.

Since the problem of (19) is also nonconvex, we handle it using SDP relaxation theory similar to that for BFA. By introducing a new variable **W** = **w** **w** ^*H*^, the problem of (19) can be transformed into

(20a)  min⁡W PAtr⁡(DAW)+PBtr⁡(DBW)+σ2tr⁡(W)

(20b)  s.t. σ2γAtr⁡(DAW)−PBtr⁡(GABW)+σ2γA≤0

(20c) PAtr⁡(GAEW)−σ2γEAtr⁡(DEW)−(σ2+PBtr⁡(GBEW))γEA≤0

(20d) PBtr⁡(GBEW)−σ2γEBtr⁡(DEW)  −(σ2+PAtr⁡(GAEW))γEB≤0

(20e)  W⪰0

(20f)  rank⁡(W)=1.

From problem (20a), it can be observed that the resulting objective function is linear, and all constraints are convex sets except the rank-one constraint. Following the SDP relaxation theory, if we drop the rank-one constraint, we can arrive at

(21) min⁡W PAtr⁡(DAW)+PBtr⁡(DBW)+σ2tr⁡(W)s.t. (20b),(20c),(20d),(20e),

which is a convex SDP, and therefore it can be efficiently solved to obtain the global optimum by the available solvers, for example, CVX [13].

It should be noted that the two schemes presented in our paper can easily be extended to the case of multieavesdropper scenario directly. When multiple eavesdroppers are present in the system, the number of the secure constraint will be two times of the eavesdropper's number for the optimization problem and the SDP relaxation method can also be applied to solve the optimization problem.

Moreover, it also can be observed that when Σ = 0, problem (11) can be degenerated into problem (19), which implies that the feasible set of problem (11) is a subset of the feasible set of problem (19) and *P*_R_^∗(BFA)^ ≤ *P*_R_^∗(BFO)^.

## 5. Numerical Results

This section will provide some simulation results to validate the effectiveness of our proposed schemes and also compare the performance of the proposed schemes with other existing schemes. In the simulations, the numerical results are obtained by solving the relaxed convex optimization problems (12a) and (20a) by using CVX tools [14].

### 5.1. Effectiveness Discussion

In this subsection, we select an example to show the effectiveness of the proposed schemes. The number of antennas *K* is 4. The uniform linear array (ULA) channel model is adopted to keep the space between successive array elements half of the carrier wavelength, where the channel vectors **h**_R_, **f**_R_, and **g**_R_ are generated in terms of the Vandermonde structure. The vector $V(\varphi )={\left[1,e^{j\theta },\ldots ,e^{j(K-1)\theta }\right]}^{T}/\sqrt{K}$, where *φ* ∈ [0°, 360°) and *θ* = −*π*sin(*φπ*/180). The directions of A, B, and E are set to be 170°, 340°, and 299°, respectively. Therefore, **h**_R_ = *V*(170°), **f**_R_ = *V*(340°) and **g**_R_ = *V*(299°).

By solving the problem in (12a) and (20a), the total transmit power consumed by the relay nodes in BFO and BFA is 20.6 dBm and 17 dBm, respectively, which apparently demonstrates that our proposed BFA consumes much less power than BFO.

**Figure 3:**
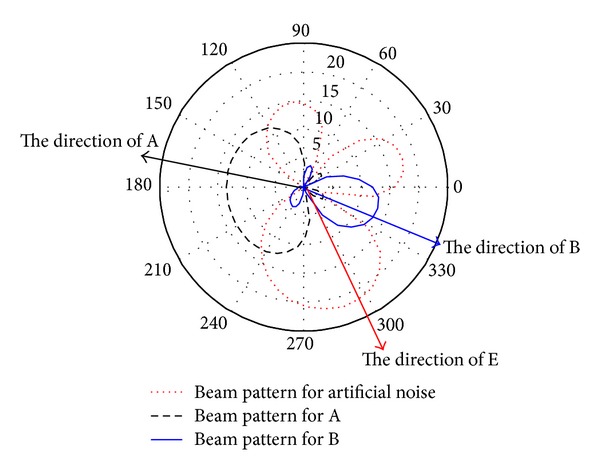
Optimal beam patterns of BFA.

**Figure 4:**
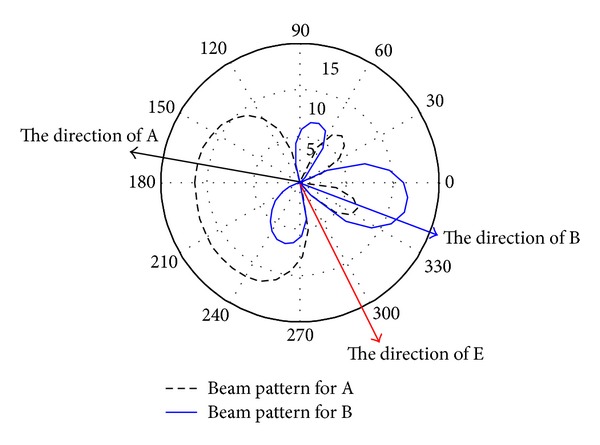
Optimal beam patterns of BFO.

To show more detail information on the beamforming vectors design, the beam patterns of w*diag⁡(**h**_R_), w*diag⁡(**f**_R_) and Σ* for problem (11) are plotted in Figure 3 and the beam patterns of w*diag⁡(**h**_R_) and w*diag⁡(**f**_R_) for problem (19) are shown in Figure 4, respectively, where w*diag⁡(**h**_R_) and w*diag⁡(**f**_R_) actually are the beam pattern for A and B, respectively, and Σ* is the beam pattern of the AN.

From Figure 3 and Figure 4, it can be seen that in the two proposed schemes, the obtained main power (information) of beam patterns w*diag⁡(**h**_R_) and w*diag⁡(**f**_R_) focuses towards A and B very well, respectively. From Figure 3 and Figure 4, it also can be observed that both w*diag⁡(**h**_R_) and w*diag⁡(**f**_R_) degrade sharply along the direction of E, which implies that, using our proposed schemes, the main power (information) can be focused towards to B and A with less leakage power towards E. So, the simulation results indicate that the major part of power for the signals is transmitted to the authorized users while only a little power of the signal is leaked to the eavesdropper.

Besides, from Figure 3, one can also see that Σ* focus its main beam power (interference) towards E, and the artificial noise power towards A and B are relatively very low. As Σ*, in fact, represents the power consumption of the designed artificial noise at the relay nodes, it demonstrates that the designed artificial noise can greatly bring down the received SINR at E while only causing very limited impact on the received SINR of the two authorized users, A and B. By doing so, secure two-way relay transmission can be achieved by using our proposed scheme.

### 5.2. Performance Comparison in terms of Infeasible Rate and Power Consumption

In this subsection, we compare our proposed BFA with BFO in terms of infeasibility rate and power consumption, where the infeasibility rate is defined as the percentage of infeasibility (%) of problems (12a) and (20a) out of 1000 simulations, which is used to evaluate the capability of the schemes in problem solving, and the power consumption is the total power consumed at the relay node. In the simulations, the channel vectors are generated as complex zeros-mean Gaussian random vectors.

**Figure 5:**
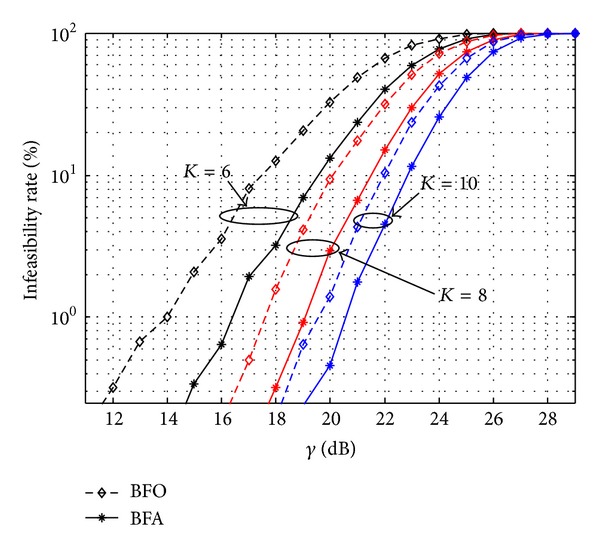
Comparison of the infeasibility rate.

**Figure 6:**
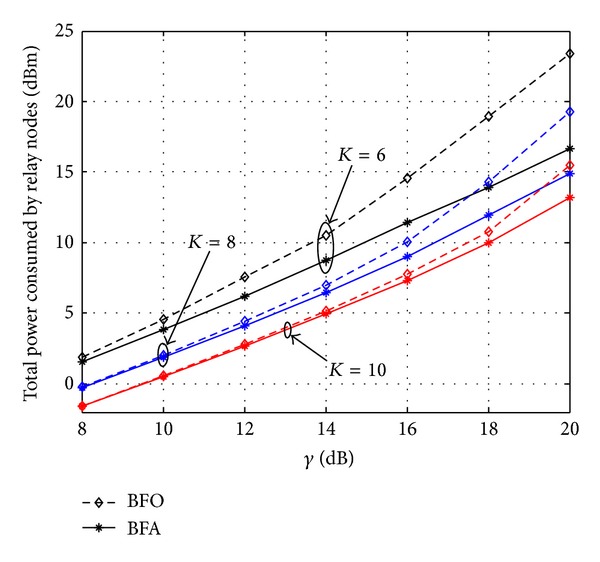
Comparison of the total consumed power.

Figure 5 plots the infeasibility rate versus *γ* when *K* are selected to be 6, 8, and 10, respectively. It can be observed that the infeasibility rate of BFA is always lower than BFO and infeasibility rates of both schemes decrease with the increase of the value of *K*.  Figure 6 plots the total consumed power *P*_R_ by the relay node versus *γ* when both of the two proposed schemes are feasible. It can be seen that the power consumption of BFA is always less than that of BFO. Moreover, the power consumption gap between the two schemes becomes gradually larger with the increase of *γ*. It also shows that the total power consumed by the relay node decreases with the growth of the number of antennas. It therefore can be stated that more relay nodes could lead to low power consumption and low infeasibility rate, and by introducing optimally designed artificial noise, secure bemforming performance can be improved.

From the simulations presented above, it can be stated that BFA always has better performance than BFO both in energy saving and feasible rate.

## 6. Conclusions

This paper studied the relay beamforming design for multiantenna two-way relay networks in the presence of an eavesdropper. We presented two beamforming methods, that is, BFA and BFO. The received SINR at the receiver was used as the QoS measurement. We formulated optimization problems for the two methods to optimally design beamforming vectors and artificial noise vector to minimize the total energy consumption. SDP relaxation theory was used to solve the problems. Simulation results demonstrated the effectiveness of our proposed schemes and showed that BFA outperforms BFO in terms of high power efficiency and low infeasibility rate, which indicated that by jointly design the artificial noise and beamforming vector, the performance secure beamforming for two-way relay systems can be greatly improved.
